# Intracranial Injection of AAV Expressing NEP but Not IDE Reduces Amyloid Pathology in APP+PS1 Transgenic Mice

**DOI:** 10.1371/journal.pone.0059626

**Published:** 2013-03-28

**Authors:** Nikisha Carty, Kevin R. Nash, Milene Brownlow, Dana Cruite, Donna Wilcock, Maj-Linda B. Selenica, Daniel C. Lee, Marcia N. Gordon, Dave Morgan

**Affiliations:** 1 University of South Florida College of Medicine, Byrd Alzheimer’s Institute, Department of Molecular Pharmacology and Physiology, Tampa, Florida, United States of America; 2 University of Kentucky Sanders-Brown Center on Aging, Department of Physiology, Lexington, Kentucky, United States of America; 3 Department of Pharmaceutical Sciences, University of South Florida College of Pharmacy, Byrd Alzheimer Institute, Tampa, Florida, United States of America; University of Florida, United States of America

## Abstract

The accumulation of β-amyloid peptides in the brain has been recognized as an essential factor in Alzheimer’s disease pathology. Several proteases, including Neprilysin (NEP), endothelin converting enzyme (ECE), and insulin degrading enzyme (IDE), have been shown to cleave β-amyloid peptides (Aβ). We have previously reported reductions in amyloid in APP+PS1 mice with increased expression of ECE. In this study we compared the vector-induced increased expression of NEP and IDE. We used recombinant adeno-associated viral vectors expressing either native forms of NEP (NEP-n) or IDE (IDE-n), or engineered secreted forms of NEP (NEP-s) or IDE (IDE-s). In a six-week study, immunohistochemistry staining for total Aβ was significantly decreased in animals receiving the NEP-n and NEP-s but not for IDE-n or IDE-s in either the hippocampus or cortex. Congo red staining followed a similar trend revealing significant decreases in the hippocampus and the cortex for NEP-n and NEP-s treatment groups. Our results indicate that while rAAV-IDE does not have the same therapeutic potential as rAAV-NEP, rAAV-NEP-s and NEP-n are effective at reducing amyloid loads, and both of these vectors continue to have significant effects nine months post-injection. As such, they may be considered reasonable candidates for gene therapy trials in AD.

## Introduction

The amyloid cascade hypothesis of Alzheimer’s disease (AD) proposes that the neurodegeneration and cognitive dysfunction characteristic of Alzheimer’s disease (AD) stem from the abnormal accumulation of amyloid-β (Aβ) within the brain [1]. Aβ is normally cleared from the brain via several mechanisms, which can be broadly divided into transport out of the CNS or enzymatic degradation of the peptide [2]. Accumulation of Aβ can therefore occur when there is an imbalance between the normal rates of production and clearance of this protein.

While excessive production of Aβ due to autosomal dominant mutations in the APP, presenilin-1 (PS1), or presenilin-2 (PS2) genes is the mechanism causing early onset, familial AD, it has been suggested that insufficient clearance of Aβ, potentially by certain Aβ-degrading enzymes may be responsible for accumulation of Aβ in cases of late onset, sporadic AD [3], [4], [5], [6]. Three of these enzymes are insulin-degrading enzyme (IDE), neprilysin (NEP), and endothelin-converting enzyme-1 (ECE-1), all of which are zinc metalloproteases normally expressed within the brain. IDE is a dimer predominantly found in the cytosol of neurons, but also exists in peroxisomes, within the plasma membrane, and is secreted from microglial cells [7]. NEP is a transmembrane glycoprotein found in both pre- and post-synaptic membranes in the nigrostriatal region and hippocampus, and is involved in the degradation of signaling peptides in the synaptic cleft. ECE-1 is also a transmembrane protein with an extracellular catalytic site, and is found in neurons, glia, and endothelial cells [8]. All three enzymes have been shown to degrade Aβ both *in vitro* and *in vivo* [5], [9], [10], [11], [12], [13].

We have previously reported on the successful over-expression of ECE-1, and the resultant reduction in Aβ load in transgenic mice due to intracranial injection of recombinant adeno-associated viral vectors (rAAV) containing the ECE-1 gene [8]. Our goal in the present study was to further explore the effects of Aβ degrading enzyme up-regulation in the brains of transgenic mice. To do this, we used rAAV vector- mediated delivery of NEP and IDE cDNA into the hippocampus and frontal cortex of APP+PS1 mice. We chose rAAV vectors because they are capable of inducing long-term transgene expression in non-dividing cells without provoking a significant inflammatory response [14]. Two forms of each protease gene were used, one that codes for the native protein (NEP-n and IDE-n), and one that codes for a truncated protein that is secreted into the extracellular space (NEP-s and IDE-s). We found that only mice injected with rAAV- NEP-s or NEP-n constructs showed a significant reduction in Aβ levels as compared to controls. This was observed in both the frontal cortex and in the hippocampus, and in both young (7.5 month old) and old (16 month old) mice. Neither IDE-s nor IDE-n caused a significant reduction in Aβ levels in either the hippocampus or anterior cortex.

## Materials and Methods

### Ethics Statement

All animal testing procedures were approved by the Institutional Animal Care and Use Committee of the University of South Florida and followed the NIH guidelines for the care and use of laboratory animals (Approval ID number A4100-01).

### Generation of NEP and IDE Gene Constructs & rAAV Vector Production and Purification

For the NEP-n and IDE-n constructs, the NEP gene (GI: 4503442) and the IDE gene (GI: 4826769) were cloned from a human GenePool cDNA library (Invitrogen) using PCR. The NEP constructs were previously described in Lebson et al (2010) [15]. Briefly, the secreted NEP (NEP-s) included amino acids 232–2313 and the GDNF signal sequence for secretion. The inactive NEP (NEP-m) contained the E585V mutation. All NEP constructs had a C-terminal haemagglutinin tag. For the IDE gene, the primers used were GAGCTCGAGACCGGTCCACCATGCGGTACCGGCTAGCGTGGCTTCTGC and GAGATCGATTCAGAGCTTAGCAGCCATGAAGTTAATATGTGG. As with NEP, a haemagglutinin (HA) tag was added to the C-terminus of IDE to distinguish the transduced gene from the endogenous IDE. The secreted/truncated form of IDE (124–3053 bp) was generated by PCR and a signal sequence (MALLVHFLPLLALLALWEPKPTQAFV) was added to the N-terminus. The human genes were examined because the human APP transgene is expressed in these mice (and thus the Aβ) and we wanted to examine if these would be useful proteases for translation into human use. The NEP-s and IDE-s were designed with the belief that a secreted form would allow for diffusion and therefore achieve a larger area of protease activity than the native membrane bound. Production and purification of rAAV vectors was performed according to the methods of Zolotukhin et al. [16]. rAAV serotype 5 was used for study 1, and rAAV serotype 9 was used for studies 2 and 3. Infectious rAAV particles were expressed as vector genomes (vg)/mL. Vector genomes were quantitated using a dot blot protocol [16].

### Study Design

Transgenic APP+PS1 mice were used for 3 independent experiments. These mice were obtained from a breeding colony at the University of South Florida, and have been maintained for >15 years. This line has been shown to have a selective increase in the level of Aβ_42_ as opposed to Aβ_40_ [17] and to develop extracellular Aβ deposits, which increase in number, size and density with aging (Gordon et al, 2002). Mice were kept under standard vivarium conditions. Intracranial injections, mice handling, and euthanasia were performed as described in Carty et al [18].

In study 1, 6 month old APP+PS1 mice were randomly assigned to three groups. The first group received unilateral, intracranial injections of rAAV vector expressing NEP-m (n = 15), an inactive form of NEP, in both the right anterior cortex (AP 2.0, Lat 2.0, DV −3.0) and right hippocampus (AP 2.7, Lat −2.7, DV −3.0). The second group received identically- placed injections of rAAV-NEP-n (n = 18), the native form of NEP, and the third group received identically placed injections of rAAV-NEP-s (n = 17), the secreted form of NEP. Mice were sacrificed at 7.5 months old, 6 weeks after the injection.

In study 2, 6 month old APP+PS1 mice received unilateral, intracranial injections of rAAV vector expressing GFP (control) in the right anterior cortex and right hippocampus (coordinates as above; n = 8). The second (n = 8) and third (n = 8) groups received identically placed injections of either rAAV-IDE-n, the native form of the IDE gene, or rAAV-IDE-s, the secreted form of the gene. The mice were euthanized at 7.5 months old, or 6 weeks post injection. In study 3, 7 month old APP+PS1 mice were split into four groups: the first group (n = 9) was untreated. The second group (n = 7) received bilateral, intracranial injections of rAAV vector expressing NEP-m in both the hippocampus and anterior cortex for a total of four injections. The third (n = 4) and fourth (n = 6) groups received injections of rAAV-NEP-n or rAAV-NEP-s, respectively. Animals were euthanized at 16 months of age, or 9 months post injection.

### Immunohistochemistry

The methods of brain fixation, cryoprotection, and sectioning are described in Carty et al [18]. For all three studies, free-floating immunochemical and histological analysis was performed on six to eight sections from each mouse brain, with the sections spanning the injection site as described in Wilcock et al [19]. IDE and NEP expression was assessed using anti-HA biotinylated rabbit polyclonal antibody (Roche, Indianapolis, IN). For all studies, Aβ was assessed using a rabbit primary anti-Aβ antibody (Gift from Dr. P Gottschal). The epitope for this antibody is N-terminal, and the antiserum does not discriminate Aβ_x−40_ vs. Aβ_x−42_. In study 3, the level of microglial activation was also assessed by using rabbit primary anti-CD45 antibodies (ThermoScientific, Rockford IL.) and anti-CD68 antibodies (AbD Serotec). Congo red staining to assess compact congophilic plaque load was performed as described in Carty et al [8].

### Imaging and Data Analysis

Stained sections were imaged using an Evolution MP digital camera mounted on an Olympus BX51 microscope at 100× final magnification (10× objective), except for the congo red staining for study 2. The sampling procedure, segmentation of positive staining and quantification of percent area of positive stain was performed as described in Carty et al [18]. The Congo red stained sections from study 2 were imaged using a Zeiss Mirax Scan 150 microscope, and identification of positive staining product and percent area of positive stain was performed using Image Analysis software (created by Andrew Lesniak, Zeiss), also as described in Carty et al [18]). Within each study and brain region, data were analyzed using one-way ANOVA statistical analysis followed by Fisher’s LSD test for individual means differences, as recommended by the computer software program StatView® version 5.0.1 (SAS Institute, Raleigh, NC). All animals in each group outlined above were used in data analysis.

### Enzyme Activity Assay

Activity of the IDE constructs was assessed using the Innozyme™ Insulysin/IDE Immunocapture kit (Calbiochem). HEK293 cells were transfected with IDE-n, IDE-s, and GFP plasmids using the Lipofectamine™ 2000 by Invitrogen protocol. Cells were harvested 48 hours later by centrifugation (5 min, 1,500×g). The cell pellets were re-suspended with 250 µL of lysis buffer (20 mM HEPES, 5 mM KCl, 1.5 mM MgCl_2,_ pH 7.5), and subjected to three freeze-thaw cycles to obtain the cell lysates. The protein concentration of each cell lysate was determined using a BCA assay (Per Pierce Protocol). Western blot was performed to confirm IDE-n and IDE-s protein expression (using an anti-HA antibody), and enzyme activity was measured. Fluorescence was measured using a BioTek spectrofluorometer plate reader at 37°C. Enzyme activity values are expressed as relative fluorescent units (RFU)/min/mg of lysate.

## Results

### Study 1

**Figure 1:**
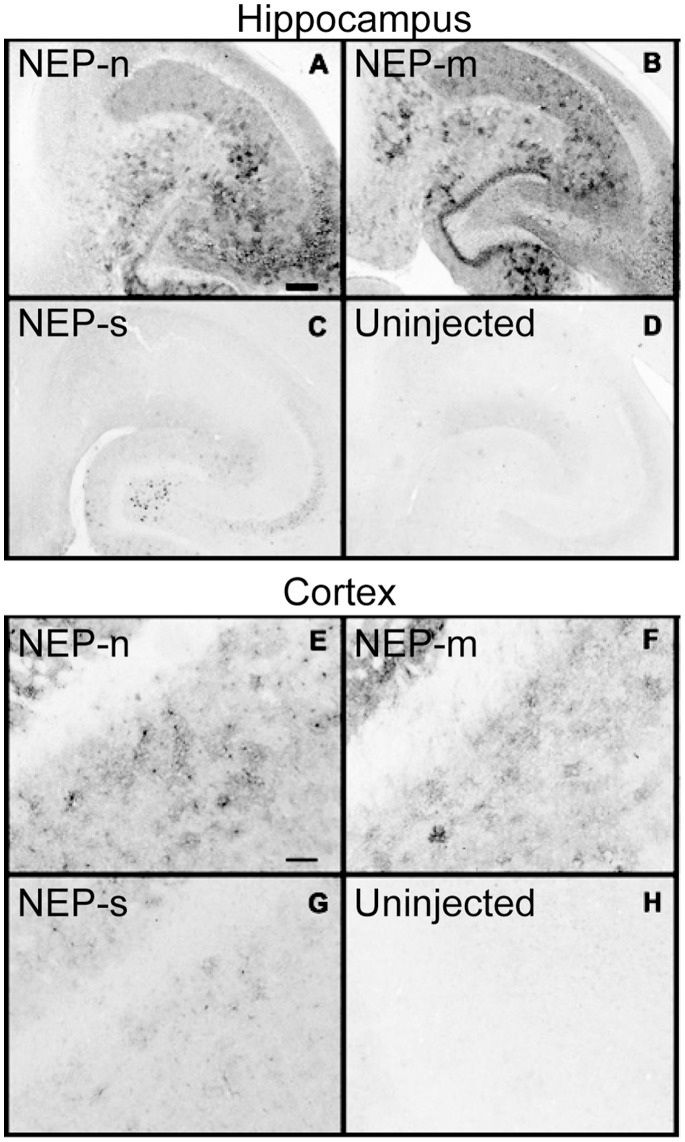
Study 1: Distribution of HA expression in the hippocampus (A–D) and frontal cortex (E–H) following intracranial administration of rAAV vectors, detected using an anti-HA antibody. Panels A & E show NEP-n treated animals; panels B & F show NEP-m treated animals; panels C & G show NEP-s treated animals. Panels D & H show no positive staining in the contralateral uninjected left hippocampus and left anterior cortex, respectively. Scale bar = 120 µm.

In this experiment, we examined the over-expression of the three rAAV vectors designed to express different versions of the human NEP gene that we described previously [15]. These included the native, membrane bound form of the enzyme (NEP-n), a secreted form of the enzyme (NEP-s), and an enzymatically deficient mutant enzyme (NEP-m). Enzyme activity was previously reported for the membrane and soluble NEPs [15]. The NEP-s enzyme was successfully secreted from cells, as evidenced by its detection in the cell media fractions from NEP-s transfected HEK293 cells (results not shown). The transgene expression of NEP was assessed six weeks after unilateral, intracranial injections into the right hippocampus and anterior cortex of APP+PS1 mice by immunostaining of mouse brain tissue with an anti-haemagglutinin (HA) tag antibody (Fig. 1).

In the ipsilateral hippocampus, anti-HA staining revealed NEP expression in all animals for NEP-n, NEP-m and NEP-s, with widespread gene expression throughout the entire hippocampus. NEP-n was detected in CA4 neurons in the hilus, in CA2 and CA3 neurons of the pyramidal cell layer of the hippocampus, and in the entorhinal cortex (Fig. 1a). Interestingly, it appears that the cell bodies in the dentate granule cell layer were not stained as intensely as the corresponding neuropil, suggesting some synaptic localization of the transfected protein. NEP-s was detected in cell bodies of CA4 neurons in the hilus, CA3 neurons, and in a small number of cells in the molecular layers of hippocampus (Fig. 1c). NEP-s expression had a more limited area of distribution, and staining was noticeably less intense than that of both NEP-n and NEP-m. It is uncertain the degree to which this reflects reduced expression, or diffusion of the soluble enzyme *in vivo* or during tissue processing. The expression pattern of NEP-m was very similar to that of NEP-n, with expression throughout the hippocampus, including the dentate gyrus and all CA regions (Fig. 1b). There was no noticeable anti-HA staining in the uninjected, contralateral hippocampus (Fig. 1d).

In the ipsilateral cortex, anti-HA staining again showed that NEP-s expression was both less intense, and limited in distribution compared to both NEP-m and NEP-n (Fig. 1). NEP-n and NEP-m expression patterns were again similar, with the greatest intensity of staining in the anterior cortex (Fig. 1e, f), and lighter staining in the striatum and corpus callosum. As in the hippocampus, NEP-n expression was largely confined to the neuropil, with less staining of the neuronal somata. NEP-s staining, though, was seen mostly in the somatic area of neurons, possibly due to packaging of the enzyme into vesicular compartments in preparation for secretion (Fig. 1g). Last, when comparing the intensity of HA immunohistochemical staining in anterior cortex to that in the hippocampus for each enzyme, the staining in the anterior cortex always appeared less intense, possibly indicating a lower level of NEP gene expression in this region. Again, there was no noticeable anti-HA staining in the uninjected, contralateral anterior cortex (Fig. 1h).

**Figure 2:**
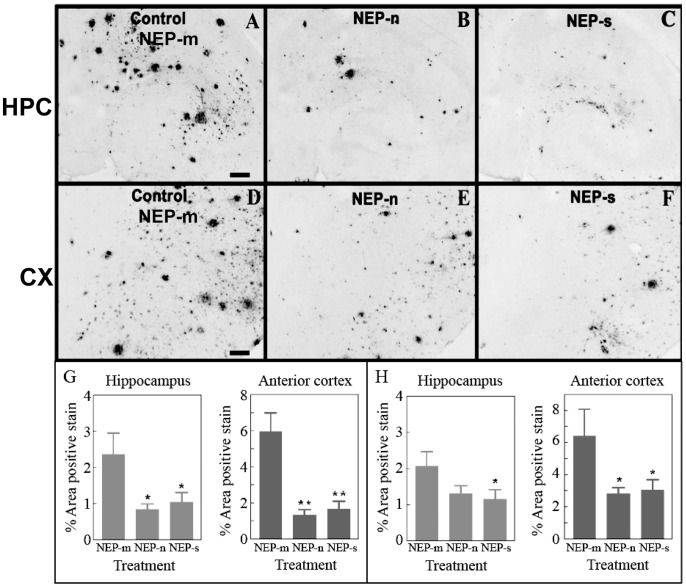
Study 1: Aβ immunostaining is observed in mice throughout both the ipsilateral hippocampus (A, B and C) and ipsilateral anterior cortex (D, E and F). Aβ staining in the hippocampus of animals that received intracranial injections of rAAV- NEP-n (B) or NEP-s (C) is reduced compared to staining in those animals that received injections of control vector rAAV- NEP-m (A). Aβ staining in the anterior cortex of mice that received intracranial injections of rAAV- NEP-s (F) or NEP-n (E) is also reduced compared to staining in mice that received control vector rAAV- NEP-m (D). Scale bar = 120 µm. Quantification of percent area of positive total Aβ staining is shown in the hemisphere ipsilateral to injection sites (G) and in the hemisphere contralateral to injection sites (H). NEP-n (n = 18), NEP-m (n = 15), NEP-s (n = 17). The (*) indicates significance compared to NEP-m with p<0.05; the (**) indicate significance compared to NEP-m with p<.001.

Next, we investigated Aβ levels of APP+PS1 mice following the NEP administration. Animals injected with the control rAAV-NEP-m showed both darkly stained compact plaques and more lightly stained, more diffuse plaques, which were distributed throughout the anterior cortex (Fig. 2d) as well as in the hippocampus (Fig. 2A). The staining was most concentrated in the molecular layers of the dentate gyrus and the CA1 region surrounding the hippocampal fissure. This staining pattern was comparable to that in age matched, untreated APP+PS1 transgenic mice [20]. Compared to mice injected with rAAV-NEP-m, mice injected with either rAAV-NEP-n or rAAV-NEP-s showed significantly less Aβ staining in both the hippocampus and anterior cortex (Fig. 2). ANOVA analysis of Aβ burden in NEP-n mice revealed a significant decrease of 78% in the anterior cortex as compared with NEP-m mice, and a significant decrease of 65% in the hippocampus as compared to NEP-m mice (Fig. 2g). Decreases in Aβ were also observed in mice that received rAAV-NEP-s. ANOVA analysis of Aβ in NEP-s mice showed a significant decrease of 60% in the anterior cortex and 56% in the hippocampus as compared with NEP-m mice (Fig. 2g). These reductions in Aβ staining were not limited to the areas ipsilateral to the injection sites – reductions were also noted in the contralateral anterior cortex and hippocampus. Quantification and ANOVA analysis of Aβ in NEP-n mice showed a significant reduction of 56% in the contralateral anterior cortex, and a reduction of 36% in the contralateral hippocampus (Fig. 2h). Similarly, analysis of Aβ in NEP-s mice showed significant decreases of 53% and 44% in the contralateral anterior cortex and contralateral hippocampus, respectively (Fig. 2h). We did not observe significant neuron staining for NEP on the contralateral side, which may suggest that we are seeing axonal transport of NEP rather than retrograde transport of the injected virus.

**Figure 3:**
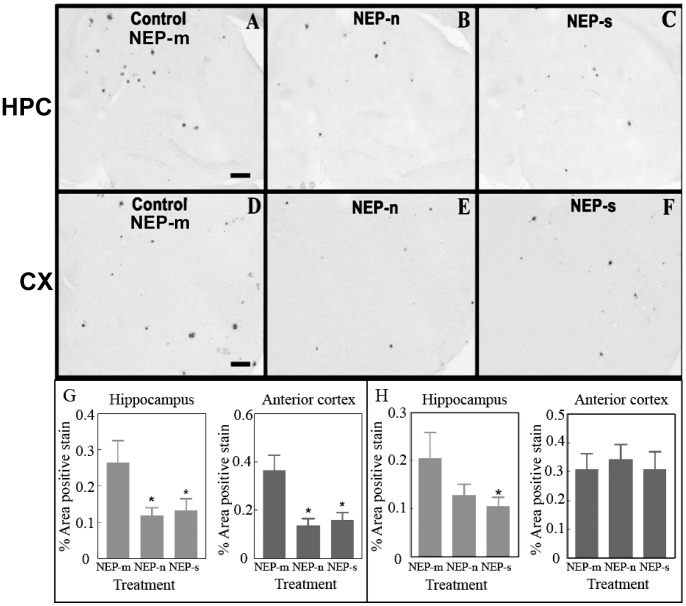
Study 1: Congophilic staining is observed in mice throughout both ipsilateral hippocampus (A, B and C) and ipsilateral anterior cortex (D, E and F). Congophilic staining in the hippocampus of animals that received intracranial injections of rAAV- NEP-n (B) or NEP-s (C) is reduced compared to staining in those animals that received injections of control vector rAAV- NEP-m (A). Congophilic staining in the anterior cortex of mice that received intracranial injections of rAAV- NEP-s (F) or NEP-n (E) is also reduced compared to staining in mice that received control vector rAAV- NEP-m (D). Scale bar = 120 µm. Quantification of percent area of positive total Congophilic staining is shown in G (hemisphere ipsilateral to injection sites) and H (hemisphere contralateral to injection sites). NEP-n (n = 18), NEP-m (n = 15), NEP-s (n = 17). The (*) indicates significance compared to NEP-m with p<0.05.

Histochemical analysis of congophilic amyloid plaques revealed that animals injected with the control rAAV-NEP-m showed positive congophilic staining patterns throughout the cortex and hippocampus (Fig. 3a, 3d), in a pattern comparable to that of age matched, untreated APP+PS1 transgenic mice (Gordon et al, 2002). Compared to mice treated with NEP-m, mice treated with NEP-n and NEP-s showed visibly less positive congophilic staining, especially in the hippocampus (Fig. 3). ANOVA analysis of congophilic load revealed that animals receiving rAAV-NEP-n showed significant reductions of 56% in both the ipsilateral hippocampus and anterior cortex as compared to mice treated with NEP-m (Fig. 3g). Mice receiving rAAV-NEP-s also had significant decreases in the compact plaque load in both the ipsilateral hippocampus (51%) and anterior cortex (57%) compared to NEP-m mice (Fig. 3g). In the contralateral hemisphere, a significant decrease as compared to NEP-m mice was only observed in the hippocampus of animals treated with NEP-s (49%; Fig. 3h). NEP-n treated mice showed a trend towards decreased congophilic staining in the contralateral hippocampus, but no significant difference as compared to NEP-m mice was observed. There were no significant decreases in congophilic staining in the contralateral anterior cortex of either NEP-n or NEP-s treated mice (Fig. 3h).

These results with NEP are similar to those observed previously with rAAV delivered ECE [8]. However it is worth noting that a construct of a soluble secreted ECE did not seem to yield an active enzyme, as we did not observe a decrease in amyloid loads in APP+PS1 mice as we do with the secreted NEP (data not shown).

### Study 2

In study 2, two rAAV vectors expressing different versions of the human IDE gene were developed in a manner similar to that used in study 1. The two versions included the native, membrane bound enzyme (IDE-n), and a secreted form of the enzyme (IDE-s). As a control, this study used a rAAV vector expressing GFP. All gene sequences were tagged with a HA peptide sequence for detection within the brain and discrimination from endogenous IDE.

Like the NEP constructs, the IDE constructs were tested prior to virus production. Constructs were transfected into HEK293 cells to evaluate the expression and the effectiveness of the signaling peptide. IDE-n transfected cell lysate, but not media, was positive for IDE protein expression by Western analysis. However, IDE-s transfected cell lysate and media was positive for IDE protein, showing effective secretion of the IDE protein due to the signaling peptide (data not shown). The cell lysate was also used to examine IDE activity. The IDE specific activity of IDE-n transfected cells was 3029 RFU/min/mg, that of IDE-s transfected cells was 166 RFU/min/mg, and that of GFP- transfected cells was 2.75 RFU/min/mg. Western analysis showed similar levels of recombinant protease expression for both forms, which indicates that the secreted form, although active, has less activity than the native IDE. This suggests that unlike NEP-s, which retained a similar activity to the native NEP [15], IDE-s may be a less effective construct.

Sections were stained for HA after administration of IDE constructs. Similar to what was observed with NEP-s in study 1, IDE expression was revealed in both IDE-n and IDE-s treated mice in the ipsilateral hippocampus and cortex. The pattern of expression of IDE-n was similar to that observed with NEP-n, as described above (data not shown). IDE-s expression had a more limited area of distribution, and staining was less intense than that of IDE-n. In the transgenic control mice and in the hippocampus and anterior cortex contralateral to the injection sites in treated mice, there was no noticeable anti-HA staining.

**Figure 4:**
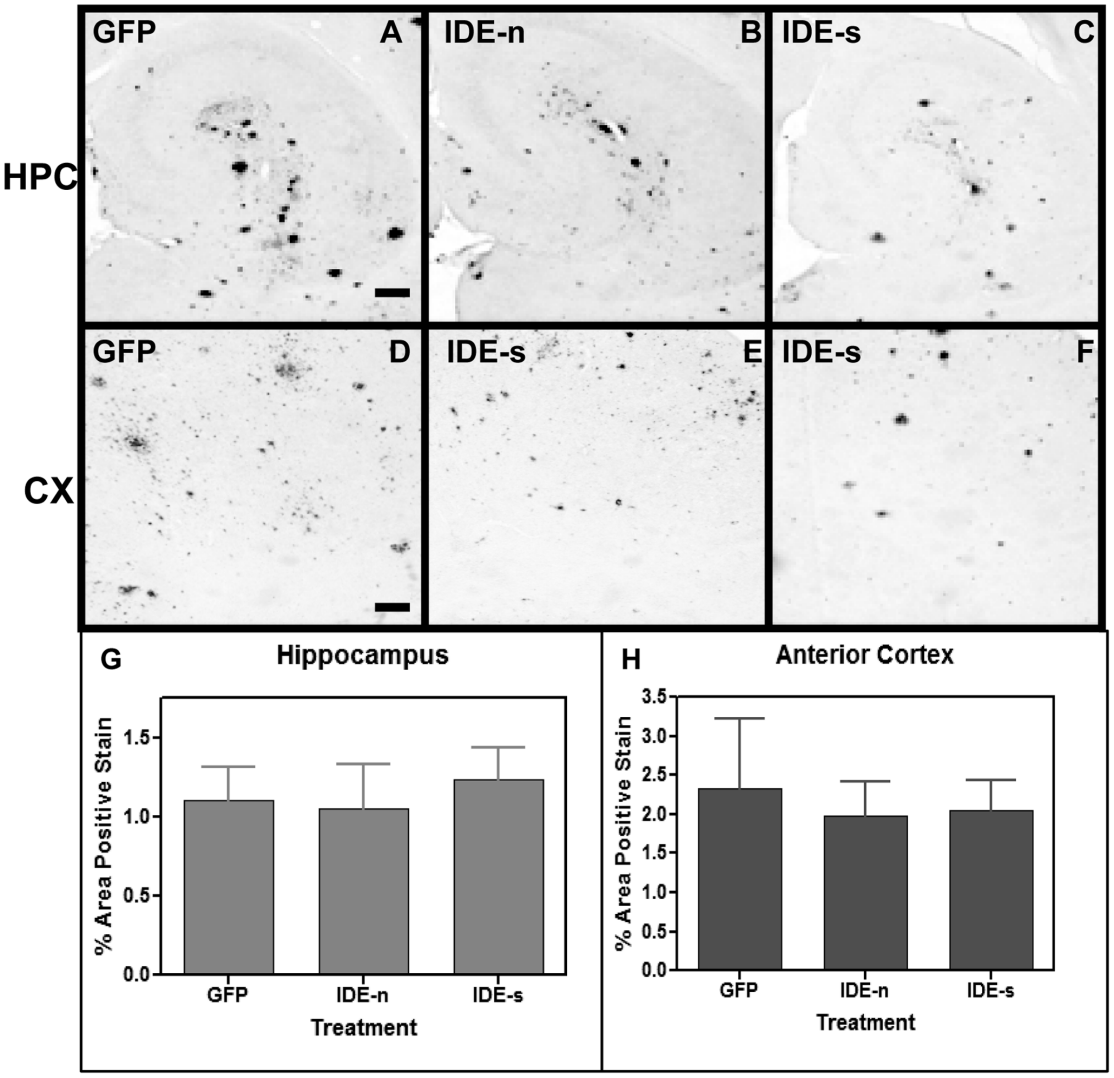
Study 2: Aβ immunostaining is observed in mice throughout both the ipsilateral hippocampus (A, B and C) and ipsilateral anterior cortex (D, E and F). Aβ staining in the hippocampus of animals that received intracranial injections of rAAV- IDE-n (B) or IDE-s (C) is unchanged compared to staining in those animals that received injections of control vector rAAV- GFP (A). Aβ staining in the anterior cortex of mice that received intracranial injections of rAAV- IDE-n (E) or IDE-s (F) is also unchanged compared to staining in mice that received control vector rAAV- GFP (D). Scale bar = 120 µm. Quantification of percent area of positive staining is shown in the hippocampus (G) and in the anterior cortex (H). No significant differences were observed. n = 8/group.

**Figure 5:**
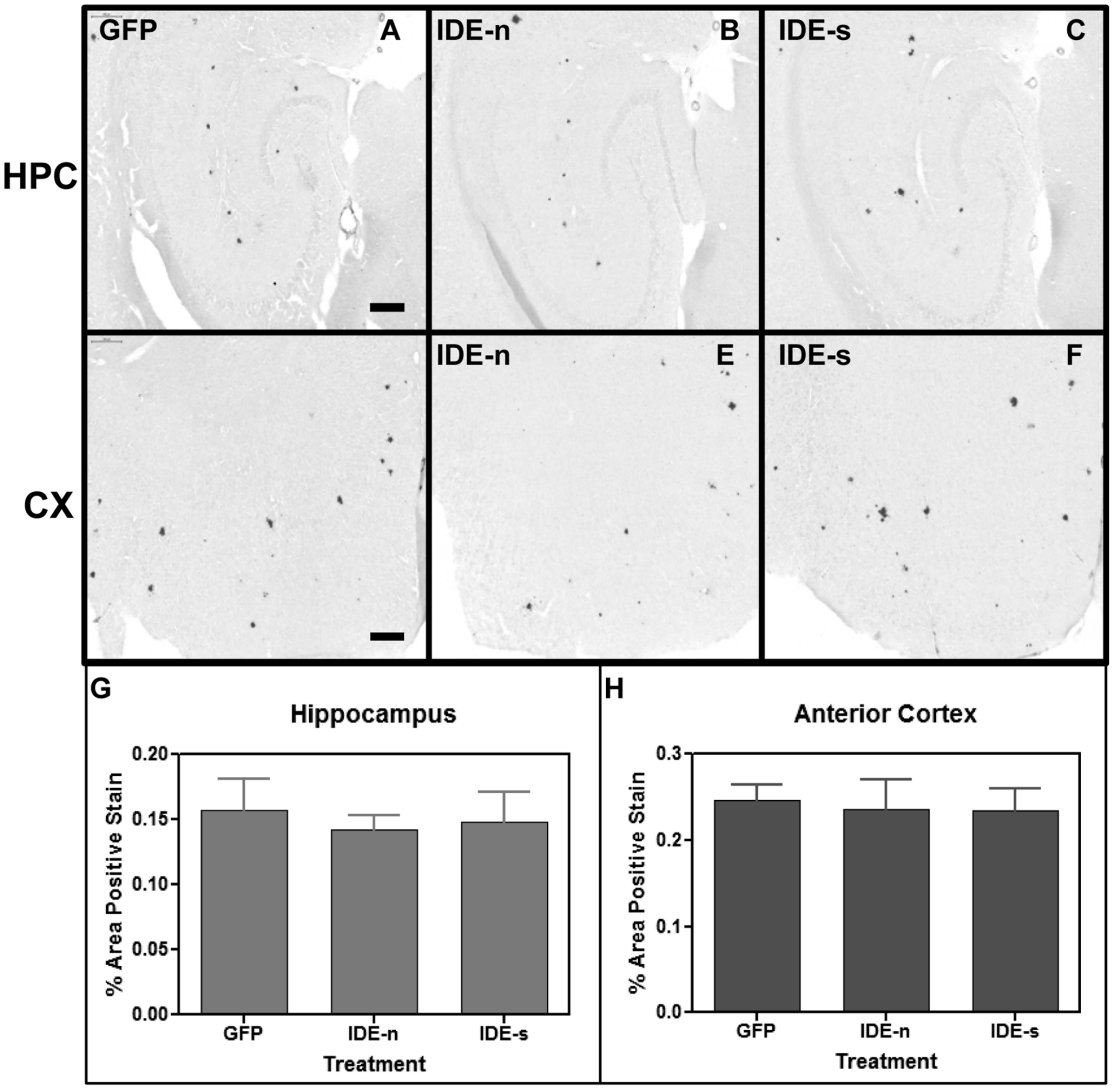
Study 2: Congophilic staining is observed in mice throughout both ipsilateral hippocampus (A, B and C) and ipsilateral anterior cortex (D, E and F). Congophilic staining in the hippocampus of animals that received intracranial injections of rAAV- IDE-n (B) or IDE-s (C) is unchanged compared to staining in those animals that received injections of control vector rAAV- GFP (A). Congophilic staining in the anterior cortex of mice that received intracranial injections of rAAV- IDE-n (E) or NEP-s (F) is also unchanged compared to staining in mice that received control vector rAAV- GFP(D). Scale bar = 200 µm. Quantification of percent area of positive total congophilic staining is shown in G and H (hemisphere ipsilateral to injection sites). No significant differences were found. n = 8/group.

Next, the effect of IDE over-expression on amyloid load in APP+PS1 transgenic mice was evaluated. In the ipsilateral hippocampus, Aβ staining patterns were very similar between the three groups (IDE-n, IDE-s, and GFP), showing comparable amounts of both darkly-stained compact plaques and lighter, more diffuse plaques (Fig. 4a–c). ANOVA analysis confirmed this, revealing no significant differences in Aβ load between the GFP, IDE-n, and IDE-s groups (Fig. 4g). In the ipsilateral anterior cortex, Aβ staining patterns were again similar between the groups (Fig. 4d–f). ANOVA analysis confirmed that there was no significant difference in total Aβ load between the IDE-n, IDE-s, and GFP groups (Fig. 4h). Similarly, Congo red staining patterns were very similar between all groups (Fig. 5), and ANOVA analysis showed no significant differences in congophilic load between the GFP, IDE-n, and IDE-s groups in either the ipsilateral hippocampus (Fig. 5g) or anterior cortex (Fig. 5h).

### Study 3

Study 3 was designed to examine a long-term over-expression of Aβ-degrading proteases. Since the IDE vectors were not successful in reducing Aβ in the short term, we focused our examination on the NEP expression vectors. Unlike the previous two studies, transgene expression was assessed nine months after bilateral, intracranial injections of the vectors into the hippocampus and anterior cortex of APP+PS1 mice.

**Figure 6:**
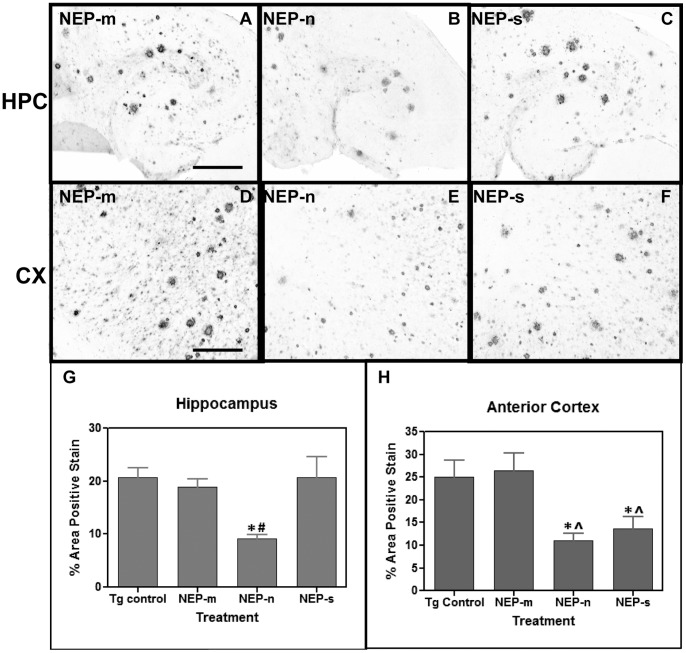
Study 3: Aβ immunostaining is observed in mice throughout both the hippocampus (A, B and C) and anterior cortex (D, E and F). Aβ staining in the hippocampus of animals that received intracranial injections of rAAV- NEP-n (B) is reduced compared to staining in those animals that received injections of control vector rAAV- NEP-m (A). Aβ staining in the anterior cortex of mice that received intracranial injections of rAAV- NEP-n (E) or NEP-s (F) is also reduced compared to staining in mice that received control vector rAAV- NEP-m (D). Scale bar = 50 µm. Quantification of percent area of positive total Aβ staining is shown in and H. The (*) indicates significance compared to NEP-m mice with p<0.05; the (^∧^) indicates significance compared to Tg control mice with p<0.05. The (#) indicates significance compared to NEP-m mice with p<0.01. Tg control (n = 9), NEP-n (n = 4), NEP-m (n = 7), NEP-s (n = 6).

Similar to study 1, animals injected with the control rAAV-NEP-m showed both darkly stained compact plaques and more lightly stained Aβ distributed throughout the anterior cortex (Fig. 6d) and hippocampus (Fig. 6a). The staining pattern in the NEP-m mice was comparable to that in age- matched, untreated APP+PS1 transgenic mice, and no significant difference was found in the level of Aβ staining between these two groups (6g, h). Compared to mice injected with rAAV-NEP-m, mice injected with rAAV-NEP-n showed significantly less Aβ staining in both the hippocampus and anterior cortex (Fig. 6b, 6e), with a significant decrease of 71% in the anterior cortex, and a significant decrease of 64% in the hippocampus as compared to NEP-m mice (Fig. 6g, 6h). Mice injected with NEP-s showed a significant decrease of 66% in Aβ load in the anterior cortex but no decrease in the hippocampus as compared to NEP-m mice (Fig. 6g, 6h).

**Figure 7:**
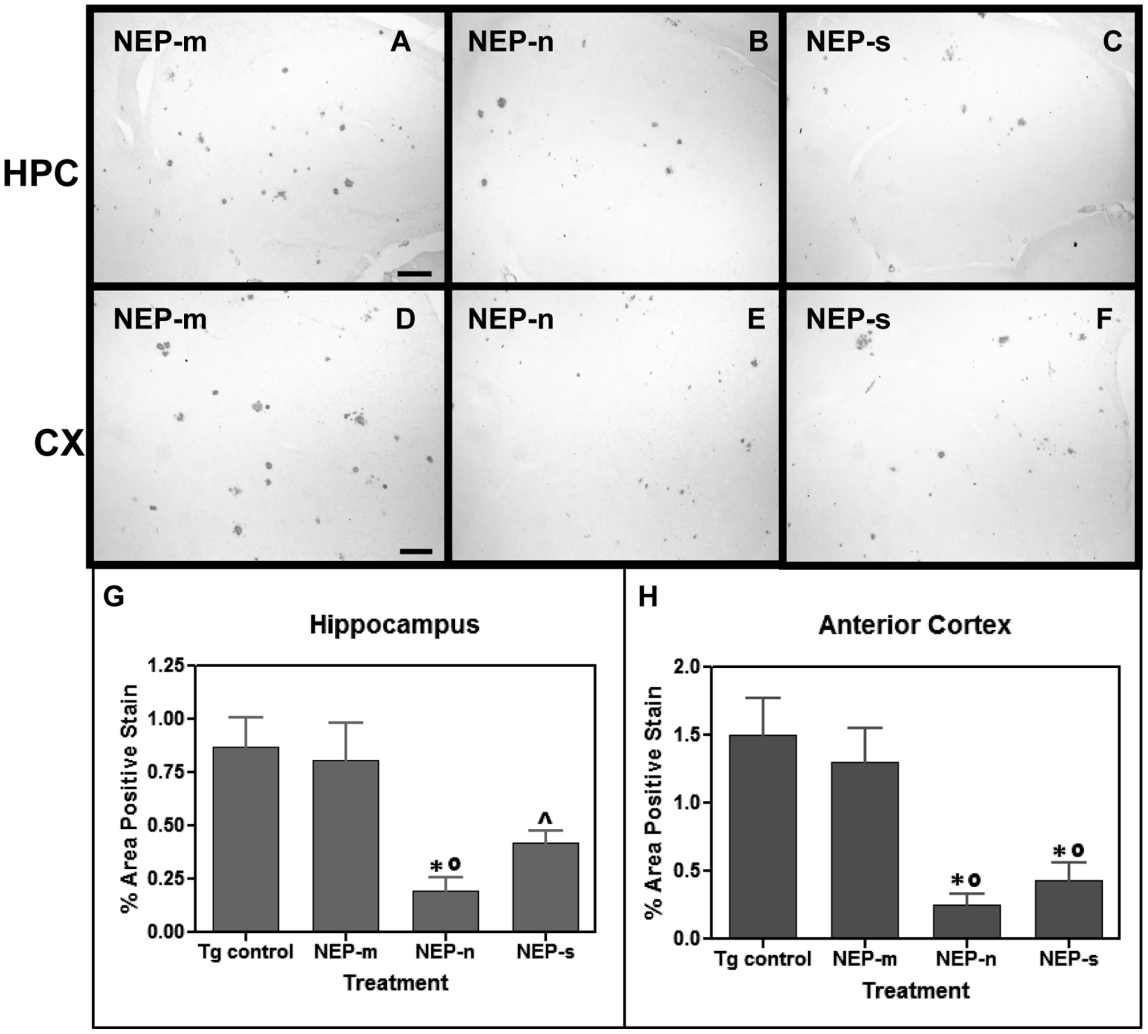
Study 3: Congophilic staining is observed in mice throughout both the hippocampus (A, B and C) and anterior cortex (D, E and F). Congophilic staining in the hippocampus of animals that received intracranial injections of rAAV- NEP-n (B) is reduced compared to staining in those animals that received injections of control vector rAAV- NEP-m (A). Congophilic staining in the anterior cortex of mice that received intracranial injections of rAAV- NEP-n (E) or NEP-s (F) is also reduced compared to staining in mice that received control vector rAAV- NEP-m (D). Scale bar = 50 µm. Quantification of percent area of positive total Aβ staining is shown in G and H. The (*) indicates significance compared to NEP-m mice with p<0.05, the (^∧^) indicates significance compared to Tg control mice with p<0.05, and the (°) indicates significance compared to Tg control mice with p<0.01. Tg control (n = 9), NEP-n (n = 4), NEP-m (n = 7), NEP-s (n = 6).

Congophilic plaque load was also analyzed nine months after bilateral, intracranial injection of rAAV-NEP-m, rAAV-NEP-n, or rAAV-NEP-s. The staining pattern in the NEP-m mice was comparable to that in age matched, untreated APP+PS1 transgenic mice, and no significant differences were found in the level of congophilic staining between these two groups. Mice treated with NEP-n showed visibly less positive congophilic staining than mice treated with NEP-m in both the anterior cortex and hippocampus (Fig. 7b, 7e). ANOVA analysis of congophilic staining revealed that NEP-n treated mice experienced a significant reduction of 76% in the hippocampus, and a significant reduction of 80% in the anterior cortex compared to NEP-m treated mice (Fig. 7g, 7h). Similarly, mice injected with NEP-s showed decreases in congophilic staining in both the hippocampus and anterior cortex. By ANOVA analysis of total congophilic staining, NEP-s mice had a non-significant decrease of 48% in the hippocampus, and a significant decrease of 67% in the anterior cortex as compared to NEP-m mice (Fig. 7g, 7h).

**Figure 8:**
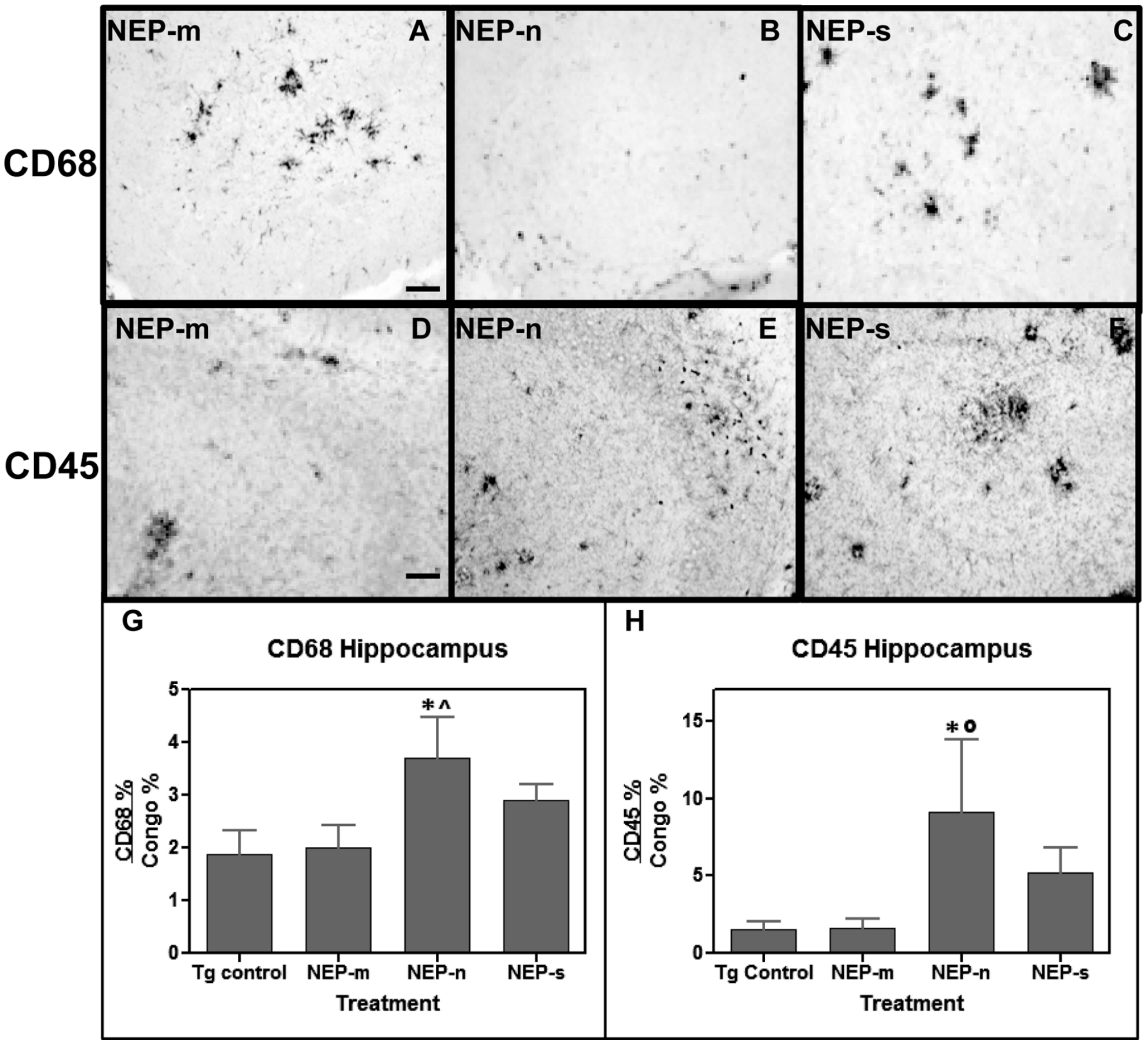
Study 3: CD68 (A-C) and CD45 (D-F) immunostaining is observed throughout the hippocampus of study mice. In the hippocampus, no significant differences are observed in the amount of total CD68 staining between the NEP-n, NEP-s, and NEP-m groups, but CD45 staining in NEP-s treated mice is greater than CD45 staining in NEP-m treated mice. Scale bar = 50 µm. Panels G and H present ANOVA analysis of the ratio of CD68 to congophilic staining, and of the ratio of quantitated CD45 to congophilic staining, respectively, in the hippocampus of study mice. The (*) indicates significance compared to NEP-m mice with p<0.05, and the (^∧^) indicates significance compared to Tg control mice with p<0.05 or p<0.01 (°).Tg control (n = 9), NEP-n (n = 4), NEP-m (n = 7), NEP-s (n = 6).

The relationship between NEP over-expression via intracranial injection of rAAV-NEP-n, rAAV-NEP-s, and rAAV-NEP-m, clearance of Aβ plaques, and immune system activation was also investigated. This was done by immunohistochemical analysis using CD45 and CD68, markers of microglial cell activation. Animals treated with rAAV-NEP-m showed both CD45 and CD68 staining throughout the hippocampus (Fig. 8a–f) and anterior cortex (not shown), in a pattern comparable to untreated transgenic mice of this age in other studies (Gordon et al, 2002). Clusters of microglia expressing these activation markers are intensely stained at the perimeter of amyloid deposits. When the fractional area is measured, there is no significant change in these markers in both the NEP-n and NEP-s expressing mice. However, differences were observed when the ratio of microglial staining:Congo red staining was calculated for the hippocampus (Fig. 8g, 8h) and the anterior cortex (data not shown). Mice treated with rAAV-NEP-n showed a significant increase (84%) in the CD68:Congo red ratio, and a significant 4- fold increase in the CD45:Congo red ratio in the hippocampus, compared to mice treated with rAAV-NEP-m (Fig. 8g, 8h). However, rAAV-NEP-n was not significantly different from the control in the anterior cortex (data not shown). Mice treated with rAAV-NEP-s trended toward increases in the CD68:Congo and CD45:Congo ratios in the hippocampus, but these were not significant (Fig. 8g, 8h). In the anterior cortex, rAAV-NEP-s showed a significant increase (1.5 fold; P<0.01) in the CD68:Congo red ratio but no difference in the CD45:congo red ratio compared to controls.

## Discussion

It is now thought that a contributing factor in cases of sporadic, late-onset Alzheimer’s disease may be a deficiency in the catabolism of the Aβ peptide by enzymes such as neprilysin (NEP) and insulin-degrading enzyme (IDE) [3], [4], [5], [6]. The potential of NEP to degrade Aβ *in vivo* was first demonstrated by Iwata *et al.* [11], who injected synthetic, radio-labeled Aβ into the CA1 region of the rat hippocampus. They showed that Aβ was most significantly degraded by NEP, and that infusion of a NEP inhibitor caused Aβ to accumulate in hippocampal fractions. Additionally, NEP- deficient mice have significant impairment in the degradation of intracranially- injected Aβ, and have significantly elevated Aβ deposition [6]. Similarly, the elevated Aβ load of APP transgenic mice can be significantly reduced when these mice are crossed with mice over-expressing NEP [10]. In humans, it has been demonstrated that NEP mRNA, protein, and activity levels are significantly reduced in high plaque areas, particularly the hippocampus and frontal cortex, in AD brains as compared to healthy controls [21], [22].

The link between Aβ and IDE was first demonstrated by Kurochkin and Goto [9], who showed that ^125^I-labeled synthetic Aβ cross-linked to a single protein within cytosol fractions from rat brain. They identified this protein as IDE, and used purified rat IDE to efficiently degrade Aβ [9]. It was subsequently shown that human IDE is also capable of degrading Aβ, and that the degradation of Aβ by IDE in cytosolic fractions from AD brains is reduced by 50% as compared to fractions from age-matched control brains [5], [23]. Membrane-bound IDE protein levels and activity are also significantly decreased in patients with AD as compared to healthy controls, and this decrease is specific to the hippocampus [24]. Studies in mice provide further evidence for the relationship between IDE and Aβ: mice homozygous for a deletion in the IDE gene on chromosome 10 have a significant decrease in Aβ degradation within brain membrane fractions [25], and the elevated Aβ load of APP transgenic mice can be significantly reduced when these mice are crossed with mice over-expressing IDE [10].

Recently, other studies using viral vectors to introduce Aβ- degrading enzymes into the brains of transgenic mouse models of Alzheimer’s disease have been performed. Marr et al. [26] have shown that intracranial injections of lentiviral vectors expressing NEP result in a significant reduction in Aβ load in 12–20 month old APP mice, and Iwata et al. [27] have achieved similar results using rAAV expression of NEP in the brains of young and old APP mice. More recently, Hong et al. [28] have shown that administration of a lentiviral vector expressing APP results in rapid Aβ deposition in mice, which can be prevented by co-administration of an HSV vector expressing NEP. Lastly, Spencer et al. [29] have shown continued over-expression of NEP and reduction of Aβ load up to six months post injection of lenti-NEP in transgenic APP mice. However, there have not been any similar studies investigating the use of viral vector expression of IDE to degrade Aβ in the transgenic mouse brain.

In the present study, our goal was to further explore the effects of Aβ degrading enzyme up-regulation via injection of viral vectors. More specifically, there were three major issues that we have attempted to address. First, the above mentioned studies on this topic incorporated the native form of the NEP gene into their respective viral vectors [26], [27], [28], [29]. The native NEP enzyme is a transmembrane protein, which may have limited access to extracellular amyloid deposits. To potentially expand the distribution of the NEP enzyme and improve Aβ clearance, we created a construct encoding a secreted form of the enzyme by removing the transmembrane domain and adding a secretion signal sequence to the N-terminus of the native NEP gene. HA was synthesized after viral transduction both in vivo (Fig. 1) and in transfected HEK293 cells. The NEP-s enzyme was successfully secreted from cells, as evidenced by its detection in the cell media fractions from NEP-s transfected HEK293 cells (results not shown). Also, the media fractions from rAAV-NEP-s transfected cells had a significantly higher specific NEP activity than the media fractions from either rAAV-NEP-m or rAAV-NEP-n transfected cells [15]. Viral constructs were expressed *in vivo*; six weeks after intracranial injection, expression of rAAV-NEP-m and rAAV-NEP-n was widespread throughout the hippocampus and anterior cerebral cortex, although the anti-HA immunostaining in NEP-s mice appeared less intense. This may be due to the diffusion of the NEP-s into the neuropil. Both enzymes resulted in a significant reduction in Aβ and Congophilic plaque burden in the ipsilateral hippocampus and anterior cortex as compared to NEP-m treated mice. However, NEP-s may exert effects over a larger volume, because NEP-s treated mice experienced slightly more reduction in Aβ and Congo red staining in the contralateral hippocampus and anterior cortex than did NEP-n mice. These results show that the ability of NEP-s to reduce amyloid load is comparable to that of the native NEP enzyme, and suggests that NEP-s may indeed be capable of degrading amyloid over a larger brain area than the native NEP enzyme.

The Aβ observed reductions on the contralateral side of the mouse brain may result from one of two reasons. Firstly, the NEP protein is axonally transported to synapses [27] and thus may travel via commissural projections to the contralateral side contributing to increased NEP activity and Aβ reduction. Alternatively, the AAV constructs may be retrogradely transported via nerve terminals at the injection site to cells on the contralateral side where the NEP would be expressed. We suspect the first alternative is most likely, as we have not previously seen significant neuronal cell body transduction on the contralateral side only diffuse staining when examining with the reporter GFP [18].

Another protease capable of degrading Aβ is IDE. Over-expression of IDE resulted in reduction in Aβ deposition [10], while knockout of IDE was associated with increased Aβ deposition in transgenic mouse models [25]. However, there have not been any studies using viral transduction methods to increase IDE expression to date. In this study, we further examined the potential therapeutic effects of the IDE enzyme by incorporating both native IDE (IDE-n) and a truncated, secreted form of IDE (IDE-s) into rAAV vectors for intracranial injection into APP+PS1 mice. Results of an *in vitro* activity analysis showed that lysates from both IDE-n and IDE-s transfected HEK293 cells displayed IDE enzyme activity and similar levels of expression on Western blot. However, the specific activity in IDE-n lysates was approximately 18-fold higher than that in IDE-s lysates. The reduced activity of the IDE-s enzyme may be due to the truncation that was performed on the native IDE gene. Song et al. have shown that deletion of C-terminal residues 1002–1019 of the IDE gene creates instability in the normal dimer interface of the enzyme, causing it to become a monomer. This resulted in a reduction of the rate of Aβ degradation to 0.25–1% the rate of wild type IDE [30]. In vivo, expression of both IDE-n and IDE-s were observed in the ipsilateral anterior cortex and hippocampus in a pattern similar to that presented for Study 1. However, neither enzyme resulted in a reduction of either Aβ or Congophilic plaque load in either brain region. These results suggest that the rAAV-IDE-n and rAAV-IDE-s constructs do not have the same therapeutic potential as the rAAV-NEP-n and rAAV-NEP-s constructs.

Lastly, we examined the long-term over expression of rAAV-NEP-s compared to rAAV-NEP-n. We observed that both NEP-n and NEP-s continued to be expressed in the APP+PS1 mouse brain nine months post-injection, as evidenced by anti-HA immunostaining. Furthermore, mice that received NEP-n experienced a significant decrease in Aβ and Congophilic plaques in each brain region as compared to both groups of control mice. Compared to NEP-n mice, mice that received NEP-s did experience equally significant reductions in Aβ and Congophilic plaques in the anterior cortex, but did not show the same degree of amyloid reduction in the hippocampus. In this brain region, NEP-s mice showed a significant reduction in Congophilic plaques as compared to the Tg control mice, but Aβ was not significantly reduced as compared to either control group.

Finally, we examined microglial activation after viral transduction. Previous studies have shown that injection of LPS causes a reduction in Aβ that is concurrent with microglial activation, and can be blocked with dexamethasone [31], [32]. Nine months after intracranial injection of either rAAV-NEP-n or rAAV-NEP-s, we investigated the level of immune activation by staining for CD45 and CD68, both markers of activated microglial cells. We observed that the ratio of CD45:Congo and CD68:Congo red were both significantly elevated in the hippocampus of rAAV-NEP-n treated animals as compared to untreated Tg and rAAV-NEP-m treated controls. Neither the CD45:Congo red nor the CD68:Congo red ratio was significantly elevated in rAAV-NEP-s treated animals. These results suggest that the native NEP enzyme, but not the secreted version, was able to sensitize the microglial reaction towards the Congophilic deposits, possibly facilitating their removal. For uncertain reasons the NEP-s appeared less capable of this activity. This may be why the NEP-n was as effective as the NEP-s in the 9 month study.

In conclusion, we further explored the efficacy of viral vector mediated introduction of Aβ-degrading enzymes at reducing the amyloid load in transgenic mice in three major ways. First, we created a rAAV vectors containing a truncated, secreted form of the NEP gene, rAAV-NEP-s, to potentially expand the brain area over which Aβ is degraded. Second, we created a rAAV vector containing IDE to evaluate whether this enzyme produces results comparable to rAAV-NEP. Third, we conducted a long-term study in which rAAV-NEP was allowed to remain in the mouse brain for nine months. Our results for these studies indicate that while rAAV-IDE does not have the same therapeutic potential as rAAV-NEP, rAAV-NEP-s and NEP-n are effective at reducing amyloid loads, and both of these vectors continue to have significant effects nine months post-injection. As such, they may be considered reasonable candidates for gene therapy trials in AD.
